# MicroRNAs in Huntington’s Disease: Diagnostic Biomarkers or Therapeutic Agents?

**DOI:** 10.3389/fncel.2021.705348

**Published:** 2021-08-06

**Authors:** Xiaoyu Dong, Shuyan Cong

**Affiliations:** Department of Neurology, Shengjing Hospital of China Medical University, Shenyang, China

**Keywords:** Huntington’s disease, microRNA, diagnosis, biomarker, therapy

## Abstract

MicroRNA (miRNA) is a non-coding single-stranded small molecule of approximately 21 nucleotides. It degrades or inhibits the translation of RNA by targeting the 3′-UTR. The miRNA plays an important role in the growth, development, differentiation, and functional execution of the nervous system. Dysregulated miRNA expression has been associated with several pathological processes of neurodegenerative disorders, including Huntington’s disease (HD). Recent studies have suggested promising roles of miRNAs as biomarkers and potential therapeutic targets for HD. Here, we review the emerging role of dysregulated miRNAs in HD and describe general biology of miRNAs, their pathophysiological implications, and their potential roles as biomarkers and therapeutic agents.

## Introduction

Huntington’s disease (HD) is a neurodegenerative disorder caused by abnormal amplification of CAG sequences in the *Huntingtin* (*Htt*) gene on chromosome 4. This results in the production of a mutant huntingtin (mHTT) protein with an abnormally long polyglutamine repeat (Jacobsen et al., 2010). The pathogenic gene, *Htt*, located on chromosome 4p16.3 and was identified in 1993 (Bates et al., 2015). HD is related to the unstable expansion of CAG triplet repeats in exon 1 of *Htt*. The normal *Htt* allele contains 6–35 CAG triplet repeats. CAG triplet expansion of 40 repeats or more is abnormal and complete penetration. Alleles with 36–39 CAG repeats are considered to have lower penetrance. Although the 27–35 CAG repeats are within the normal range, they are considered to be intermediate or unstable alleles that may extend or contract during reproduction. The main clinical symptoms of HD are usually classified into three categories: motor symptoms, psychiatric disorders, and cognitive dysfunction (Sheikh and Guerciolini, 2019). The typical dyskinesia of the disease is chorea-like involuntary movement. Approximately 90% of patients with HD experience this typical dyskinesia, but most of the patients’ motor symptoms are a combination of various dyskinesias, including chorea-like symptoms, dystonia, ataxia, and Parkinson’s syndrome (Heo and Scott, 2017). Psychiatric symptoms in patients with HD may appear earlier than dyskinesia, and patients may also experience anxiety and irritability (Goh et al., 2018). In addition, the cognitive impairment of HD is mainly executive dysfunction, and some studies have shown that the cognitive impairment could be worsen with the increase in the repeat length of the CAGs and the patient’s age (Bayliss et al., 2019).

Currently, the molecular mechanism of HD remains unclear; however, there are two main hypothesis: wild-type Htt (*wtHtt*) loss-of-function and mutant Htt (*mHtt*) gain-of-toxicity (Tellone et al., 2019). The *wtHtt* plays an important role in embryonic development; it is antiapoptotic, regulates gene transcription in neurons and synaptic transmission, and promotes vesicle transport function (Romo et al., 2018; Orozco-Diaz et al., 2019; Smith-Dijak et al., 2019). The loss of the normal physiological functions of *wtHtt* is involved in the pathogenesis of HD. The *mHtt* also produces additional toxic pathogenic effects, including adverse effects on apoptosis, gene transcription, axonal transport, synaptic transmission, the ubiquitin-proteasome system, and calcium ion signal transduction (Hamilton et al., 2019; Wanker et al., 2019). Although the precise mechanisms underlying HD pathogenesis have not yet been fully elucidated, it is known that transcriptional dysregulation is associated with this disease. Thus, the regulation of transcriptional regulators has been considered to be a key pathogenic mechanism in HD (Tabrizi et al., 2019).

Although some breakthroughs have been made in the study of HD pathogenesis, there is still a lack of effective approaches to treat HD. Currently, empirical symptomatic supportive therapy is the main treatment method. Antipsychotic drugs such as butbenazine or olanzapine can be used to control the chorea. Antidepressants may improve the symptoms of depression, and psychotherapy is mainly expected to alleviate cognitive dysfunction. However, these therapeutic strategies failed to meet clinical expectations (Fritz et al., 2017). Induction of mutant IT-15 gene silencing therapy can fundamentally reduce the formation of the mHTT protein to achieve the therapeutic effect. Non-specific partial inhibition of *wtHtt* and *mHtt* gene expression was effective in animals with HD, and no apparent adverse effects were detected. However, potential safety risks could not be ruled out after application in humans (Wild and Tabrizi, 2017). The optimal treatment strategy is to target specific alleles for gene interference, which selectively inhibit the expression of the *mHtt* gene but have no effect on the *wtHtt* gene (Shannon, 2020).

MicroRNAs (miRNAs) are a class of evolutionarily conserved endogenous non-coding short RNAs. By binding to the 3′ untranslated region (UTR) of the target mRNA, the expression activity of the target gene is negatively regulated by miRNAs at the post-transcriptional level to inhibit the translation or degradation of the target mRNA (Tafrihi and Hasheminasab, 2019). The miRNAs regulate the expression of approximately 90% of genes in the body and are involved in cell proliferation, development, and senescence (Ying et al., 2018). Studies have shown that miRNAs are involved in the early differentiation, development, and function of neurons (De Pietri Tonelli et al., 2008). Targeted regulation of specific miRNA expression may provide new hope for the treatment of HD. Previous studies have shown that miR-9/miR-9 significantly decreased in the brain during the progression of HD disease, which interacted with HTT by regulating the expression of repressor element-1 silencing transcription (REST) (Packer et al., 2008). In addition, miR-22 has a potential protective effect on neurons, and it has been confirmed that miR-22 could delay the progression of HD by mediating neuronal synthesis and survival (Jovicic et al., 2013).

In this review article, we introduce the canonical miRNA biogenesis pathway and miRNA function and describe the most relevant brain-specific miRNAs associated with HD. Potential biomarkers involved in HD diagnosis and the use of miRNA-based therapeutic strategies are also highlighted.

## Biosynthesis and Function of miRNAs

The miRNAs bind to complementary sequences in the UTR of target genes to inhibit their cleavage, degradation, or translation (Hajjari et al., 2017). In the nucleus, miRNA genes are transcribed into initial miRNAs (pri-miRNAs). The pri-miRNAs are processed by Drosha and Dicerase into precursor miRNAs (pre-miRNAs), which are approximately 70 nucleotides with a stem-ring structure. Exportin 5 and Ran GTPase transport the pre-miRNAs to the cytoplasm. Dicerase then cleaves the pre-miRNAs into double-stranded miRNAs of approximately 21 nucleotides. The double strands are then broken down into two single-stranded molecules, one of which degrades, and the other binds to the Argonaute proteins to form the RNA-induced silencing complex (RISC) (Yates et al., 2013). RISC binds to the 3′-UTR of target genes. Incomplete complementation of the miRNAs and target genes leads to either target mRNA degradation or inhibition of translation (Kim et al., 2016). By binding to the 3′-UTR of the target mRNA, the expression activity of the target gene is negatively regulated by the miRNAs at the post-transcriptional level, and the translation of the target mRNA is inhibited (Miranda et al., 2006).

The miRNAs are involved in nearly all biological processes, including development, proliferation, inflammation, and apoptosis (Kawahara, 2014). In addition to being intracellular, miRNA is also present in the peripheral circulation (Mori et al., 2019). By detecting the sequence, structure, type, and number of miRNAs in peripheral blood, we can understand the physiological status of an organism and the type and extent of the disease. Moreover, the detection method is simple, easy, and non-invasive, which is ideal for early screening (Majdi et al., 2016; Li et al., 2017). In recent years, it has been found that abnormal expression of miRNAs plays an important role in HD pathogenesis (Langfelder et al., 2018). The potential of miRNAs as biomarkers for diagnosis and prognosis is gradually being recognized and applied to clinical practice. Gene silencing strategies targeting miRNAs have also been tested in animal models of HD (Pfister et al., 2018).

### The miRNAs in HD

An increasing number of studies have shown that miRNAs are dysregulated in HD (Soldati et al., 2013; Kocerha et al., 2014; Reynolds et al., 2018). Sinha et al. (2012) showed that 54 miRNAs were differentially expressed in the postmortem brains of patients with HD, which included 30 miRNAs with high expression and 24 miRNAs with low expression. Among these differentially expressed miRNAs, 26 were regulated by the REST, TP53, and E2F1 transcription factors (Sinha et al., 2012). Moreover, Johnson and Buckley (2009) found that some critical neuronal miRNAs (e.g., miR-9, miR-124, and miR-132) were downregulated in the brains of HD patients and mouse models, potentially disrupting mRNA regulation and neuronal function. By using miRNA microarray analysis, Lee et al. (2007) showed full expression profiles of miRNAs in three HD mouse models. The R6/2 HD transgenic mice expressed the N-terminal exon 1 of a human *mHtt* (Ehrnhoefer et al., 2009), while the YAC128 transgenic mice expressed a full-length *mHtt* that replicated the one found in patients with HD. The authors showed that nine miRNAs (miR-22, miR-29c, miR-128, miR-132, miR-138, miR-218, miR-222, miR-344, and miR-674^∗^) were downregulated in YAC128 and R6/2. Chronic 3- nitropropionic acid (3NP) administration inhibits mitochondrial succinate Ca^2+^ homeostasis and dehydrogenase complex II, which induces striatal neurodegeneration due to mitochondrial dysfunction. In the 3NP-induced rat model of HD, the miRNA profile did not overlap with that of the transgenic mice, which may because *mHtt* modulated some aspect of the HTT activity in extra-mitochondrial energy metabolism rather than causing a direct impact on the mitochondrion (Lee et al., 2007). Accordingly, mHTT seems to compromise both the level and function of miRNAs. The mechanism by which mHTT alters miRNA biogenesis warrants future studies. HD is characterized by selective neuronal vulnerability, and neurons in the striatum and deep layer cortical neurons are the most vulnerable to degeneration; in contrast, neurons in other areas of the brain (such as the cerebellum) are relatively resistant to cell death induced by mHTT (Vonsattel and Difiglia, 1998). By analyzing the expression of miRNAs in the different regions of the brain of HD model mice with increasing CAG length in the endogenous *Htt*, Langfelder et al. (2018) found that the differential expression of miRNAs was most apparent in the striatum (159 differentially expressed miRNAs) followed by the cerebellum (102 differentially expressed miRNAs), hippocampus (51 differentially expressed miRNAs), and cerebral cortex (45 differentially expressed miRNAs). They further showed that miR-212, miR-132, miR-128, and miR-218 might be significantly associated with Htt CAG repeat expansion, thus suggesting that these miRNAs may be involved in the differential sensitivity to CAG length expansion (Langfelder et al., 2018).

**FIGURE 1:**
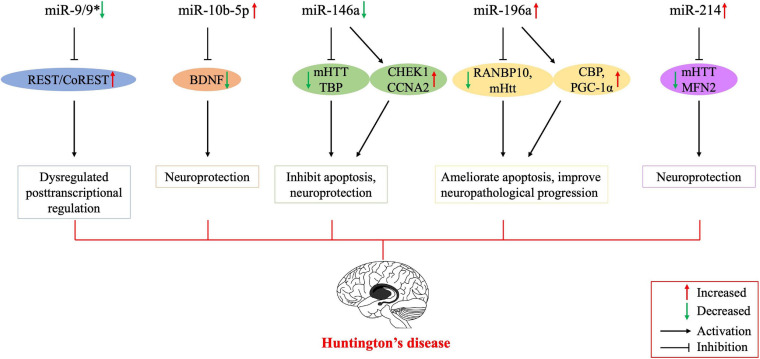
Possible mechanisms of the involvement of dysregulated microRNAs in the pathogenesis of Huntington’s disease. Several studies have been conducted on Huntington’s disease to regulate the expression of miRNAs for therapeutic purposes, and their results encourage further molecular research on all major neurodegenerative diseases, including Huntington’s disease. BNDF, brain-derived neurotrophic factor; CBP, cyclic AMP-response element-binding (CREB) protein; CCNA2, cyclin A2; CHEK1, checkpoint kinase 1; mHTT, mutant huntingtin; PGC-1α, peroxisome proliferator-activated receptor-γ coactivator-1α; MFN2, mitofusin2; REST, repressor element 1-silencing transcription factor; RANBP10, RAN binding protein 10; TBP, tata binding protein. *indicates the passenger strand of miR-9.

The miRNAs have also become a research hotspot for potential therapeutic strategies for HD that would alter their expression. Jovicic et al. (2013) demonstrated that miR-22 has various anti-neurodegenerative properties; it can inhibit apoptosis and HD-related mRNA expression. Overexpression of miR-22 inhibited neurodegeneration in the striatum and cortex due to *mHtt* fragments and improved neuronal viability in an *in vitro* HD model (Jovicic et al., 2013). Moreover, overexpression of miR-27a reduced mHTT aggregation in HD cells by modulating multidrug resistance protein-1 function (Ban et al., 2017). Liu et al. (2015) found that miR-124 could slow down the progression of HD by affecting neuronal differentiation and development. Below, we have detailed the most recent studies of miRNAs associated with HD pathology and dysregulated in this disease (Figure 1 and Table 1).

**TABLE 1:** Some of the miRNAs most commonly associated with HD.

**miRNA**	**Samples (P/HC)**	**CAG repeat (P/HC)**	**Brain region**	**Expression levels**	**Significance in HD pathophysiology**	**References**
miR-9	19/7	37-46/16-21	BA4 cortex	Down-regulated	Participation in transcriptional dysregulation, target of REST in normal condition	Packer et al., 2008
miR-10b-5p	26/36	44.6±2.9/NA	BA 9 cortex	Up-regulated	Promotes striatal involved in HD	Hoss et al., 2015a
	12/9	NA	Prefrontal cortex	Up-regulated	Regulates BNDF expression in normal condition	Müller, 2014
miR-146a	STHdh cells	111/7	NA	Down-regulated	Rescue the abnormalities of HD in cell cycle and apoptosis, regulates HTT and TBP expression in cell culture	Sinha et al., 2010, 2011
miR-196a	15/16	44.9±1.3/NA	Plasma	Up-regulated	Anti-cytotoxicity and apoptosis, regulates CBP, PGC-1α expression in HD	Cheng et al., 2013; Chang et al., 2017
miR-214	STHdh cells	111/7	NA	Up-regulated	Suppress aggregates of mHtt, regulates MFN2 expression in cell culture	Bucha et al., 2015

*BA, Brodmann’s area; BNDF, brain derived neurotrophic factor; CAG, cytosine, adenine, guanine; CBP, cyclic AMP-response element-binding (CREB) protein; HC, healthy controls; HD, Huntington’s disease; NA, Not Available; P, patients; PGC-1α, peroxisome proliferator-activated receptor-γ coactlvator-1α; MFN2, mitofusin2; REST, repressor element 1-silencing transcription factor; TBP, Tata Binding Protein.*

### The miR-9

miR-9 is encoded by three different genes and is specifically expressed in neural cells. The miR-9 is also one of the most frequently altered miRNAs and is significantly downregulated in HD (Johnson and Buckley, 2009). Dysregulation of the REST transcription factor is the most understood molecular mechanism for neurodegeneration involved in HD (Ooi and Wood, 2007). REST contains a miR-9 recognition element. miR-9 also processes the original transcriptional sequence, which can be occupied by the REST (Van Den Hove et al., 2014). Packer et al. (2008) predicted target sites for miR-9 in the 3′-UTR of REST mRNA. Moreover, miR-9^∗^ possesses a predicted site in the 3′-UTR of CoREST. In postmortem HD brain samples, bifunctional brain-enriched miR-9/9^∗^ was also validated, which targets the REST-CoREST complex. A subsequent study on the importance of miR-9^∗^ derived from peripheral leukocytes of patients with HD found that miR-9^∗^ expression levels were significantly lower in these patients and the downregulation may be a signature of neurodegeneration (Chang et al., 2017).

### The miR-10b-5p

miR-10b-5p is located in the HOXD cluster and targets HOXD4. Hoss et al. (2014) found that miR-10b-5p expression was upregulated in PC12 Q73 cells and enhanced cell survival in the presence of an apoptosis-inducing compound. They surmised that increased miR-10b-5p might play a pathological role in the expanded polyglutamine repeat of HD and was associated with the pathology of this disease. Reduced BDNF expression is involved in the neuronal dysfunction and death observed in HD. BDNF can be post-transcriptionally controlled by upregulation of miR-10b-5p. Müller (2014) suggested that miR-10b-5p upregulation in HD had a neuroprotective effect on the response to mHTT, presumably through regulating BDNF expression. Hoss et al. (2016) demonstrated that miR-10b-5p was markedly increased in HD compared to that in controls and was negatively associated with the age of HD onset, with higher miR-10b-5p levels corresponding to an earlier age of onset. The authors also determined that miR-10b-5p levels were significantly associated with CAG length in postmortem HD brain tissue (Hoss et al., 2015a). Furthermore, they found that miR-10b-5p was elevated in the plasma of individuals with HD, which might be a promising clinical diagnostic marker to predict the age and severity of HD onset (Hoss et al., 2015b).

### The miR-146a

miR-146a is a major regulator of the NF-κB pathway. Jayadev et al. (2013) and Juzwik et al. (2019) identified miR-146a as a negative regulator of the monocyte proinflammatory response. Ghose et al. (2011) found that miR-146a expression was decreased in STHdh (Q111)/Hdh (Q111) cells, and the low expression was due to the low activity of the p65 subunit of NF-κB (RelA/NF-κB). The miR-146a can also target human and mouse *Htt* gene. Sinha et al. (2011) suggested that this regulatory relationship might provide a new mechanism for the modulation of HD. In addition, decreased miR-146a expression can increase the expression of CHEK1 and CCNA2, which may rescue the cell cycle and apoptotic abnormalities in STHdh (Q111)/Hdh (Q111) cells. The miR-146a may also target Tata binding protein (TBP), and dysregulated miR-146a may contribute to HD pathogenesis by targeting TBP (Sinha et al., 2010). Das and Bhattacharyya (2015) suggested that heat shock factor 1 (HSF1) could regulate miR-146a and suppress mHTT aggregates in HD cells. Therefore, miR-146a may be involved in the pathogenesis of HD through a variety of mechanisms, and thus, regulating the expression of miR-146a may be a potential approach to treat HD through a variety of pathways (Das et al., 2015).

### The miR-196a

Elevated miR-196a expression levels have been observed in both animal and human HD brains (Chang et al., 2017). Kunkanjanawan et al. (2016) demonstrated that miR-196a overexpression could ameliorate cytotoxicity and apoptosis. It could also improve mitochondrial morphology and function in HD cells by upregulating CBP and PGC-1αα (Kunkanjanawan et al., 2016). Higher expression of RAN binding protein 10 (RANBP10) in HD transgenic mouse brains may exacerbate the pathological aggregates in HD. The miR-196a can suppress the expression of RANBP10 by binding to its 3′-UTR. Moreover, miR-196a can enhance neuronal morphology by suppressing RANBP10 expression (Her et al., 2017). By using different HD models, Cheng et al. (2013) discovered that upregulated miR-196a could suppress *mHtt* expression in the brain and inhibit the neuropathological progression of the disease. Furthermore, miR-196a-5p was shown to have a significant relationship with CAG repeat size and onset of age in HD patients (Hoss et al., 2015a). The miR-196a-5p is likely to exert a neuroprotective effect in HD, and future research may focus on its potential to treat HD. In addition, because its expression level may be related to the CAG repeat size and age of onset in HD patients, its potential as a diagnostic biomarker for HD is also worthy of attention.

### The miR-214

miR-214 targets the *Htt* gene, and its expression is increased in HD cell models (Sinha et al., 2011). Kozlowska et al. (2013) confirmed an interaction between miR-214 and the 3′-UTR of *Htt*. While *wtHtt* did not affect miR-214 or β-catenin expression, *mHtt* mediated β-catenin downregulation by upregulating miR-214 (Ghatak and Raha, 2018). HSF1-regulated miR-214 expression can suppress mHTT aggregation in an HD cell model (Das and Bhattacharyya, 2015). Bucha et al. (2015) showed that increased miR-214 expression in HD cells could target mitofusin2 (MFN2), thereby altering mitochondrial morphology and deregulating the cell cycle. Therefore, miR-214 may be a potentially critical node for therapeutic intervention in the pathogenesis of HD.

## The miRNAs as Biomarkers in HD

HD is a slow-progressing, inheritable neurodegenerative disorder. Its diagnosis mainly depends on family history and genetic testing. Although HD is an untreatable disease, biomarkers may provide early diagnostic clues or reflect disease progression and treatment response.

There has been some progress in the study of HD diagnostic biomarkers in recent years (Majid et al., 2011). The direct quantification of mHTT itself shows its promise as a disease-related biomarker (Weiss et al., 2009). Due to the accumulation of N-terminal fragments, mHTT levels increase with the progression of the disease, and mHTT concentration is correlated with “CAG–age product” (CAP) score and brain atrophy rate, indicating a potential functional correlation (Moscovitch-Lopatin et al., 2010; Weiss et al., 2012). If the ongoing work to further improve detection methods is successful, it will be possible to accurately quantify the level of mHTT protein in CSF, similar to the current use of amyloid beta peptide in Alzheimer’s disease (Blennow et al., 2010). However, a more valuable method may be to identify specific post-translational modifications or abnormal conformations of HTT that are associated with disease pathogenesis (Ross et al., 2014). The concentration of plasma neurofilament light chain (NfL) is significantly increased in patients with HD and is closely associated with age and CAG repeat length (Coarelli et al., 2021). Cerebrospinal fluid (CSF) NfL appears to be a more sensitive marker than plasma NfL to monitor disease progression, and it has also been shown to increase significantly when carriers come close to the age of expected disease onset (Scahill et al., 2020). However, changes in NfL levels are not limited to HD but are also observed in other neurodegenerative diseases such as Alzheimer’s disease, Parkinson’s disease, and amyotrophic lateral sclerosis, thus making NfL less specific for the diagnosis of HD. Leukocyte telomere length (LTL) values were observed to decrease significantly in patients with HD, and an inverse relationship was found between the mean LTL value and CAG repeat number in pre-HD patients. Thus, LTL might be a reliable biomarker to track HD progression. However, a well-designed follow-up study would be useful to verify the actual relationship between LTL and HD onset (Scarabino et al., 2019). Levels of the proinflammatory cytokine IL-6 have also been reported as a biomarker to diagnose HD (Chang et al., 2015); however, the sensitivity and specificity of the diagnosis remain to be confirmed. HD is a genetically confirmed clinical diagnosis. Predictive testing is available; however, it should be done with caution in patients who are at risk for the disease but have no clinical disease expression (Tibben, 2007). Almost a quarter of carriers may experience adverse events in the first year after disclosure (Almqvist et al., 2003). Carriers may be more pessimistic when they reach the expected age of onset (Frank and Jankovic, 2010). MiRNAs in the peripheral circulation have been extensively investigated as biomarkers for early diagnosis and monitoring of disease progression (Pan et al., 2016). In addition, changes in miRNAs levels may be associated with disease prognosis, such as Alzheimer’s disease (Zhao et al., 2020).

Previous studies have shown that miRNAs secreted in small vesicles or non-vesicles (e.g., peripheral blood, serum, plasma, saliva, and urine) can bind to proteins or other molecules. For example, miRNAs in plasma can bind to high-density lipoprotein to form stable structures in peripheral blood (Noren Hooten et al., 2013). Thus, miRNAs in the peripheral circulation may be important biomarkers for detecting HD because they can exist stably outside cells. The most important research findings for the potential role of miRNAs as biomarkers in HD patients are summarized below and in Table 2.

**TABLE 2:** Circulating miRNAs as biomarkers in HD.

**miRNA**	**Samples (P/C/HC)**	**CAG repeat means (P/PR/HC)**	**Sex M: F (P/PR/HC)**	**Age means (P/PR/HC)**	**Source**	**Expression in pre-HD/HD patients**	**References**
miR-22-5p, miR-30d-5p, miR-128, miR-130b-3p, miR-222-3p, miR-223-5p, miR-223-3p, miR-338-3p, miRNA-361-5p, miR-425-5p	15/NA/7	41.73/NA/NA	NA	NA	Plasma	Increased in HD	Diez-Planelles et al., 2016
miR-135b-3p, miR-140-5p, miR-520f-3p, miR-3928-5p, miR-4317, miR-8082	15/30/15	42.14/42.3/20.7	5:10/15:15/7:8	55.5/40.8/45.4	CSF	Increased in Pre-HD	Reed et al., 2018
miR-10b-5p, miR-486-5p	26/4/8	NA	11:15/1:3/3:5	53.0/42.5/46.1	Plasma	Increased in HD	Hoss et al., 2015b
miR-9*	36/8/28	46.42/44.13/NA	20:16/3:5/17:11	45.6/29.8/42.0	Peripheral leukocyte	Decreased in HD	Chang et al., 2017
miR-34b	16/11/12	44.5/42.0/NA	8:8/4:7/5:7	51.0/40.0/49.0	Plasma	Decreased in Pre-HD	Gaughwin et al., 2011

*CAG, cytosine, adenine, guanine; HC, healthy controls; HD, Huntington’s disease; NA, Not Available; P, patients; PR, pre-manifest HD.*

Diez-Planelles et al. (2016) investigated differences in miRNA levels between 15 symptomatic patients with 40–45 CAG repeats in the *Htt* gene and seven healthy controls. They identified 168 altered circulating miRNAs in the symptomatic HD patients. Specifically, miR-22-5p, miR-30d-5p, miR-128, miR-130b-3p, miR-222-3p, miR-223-5p, miR-223-3p, miR-338-3p, miR-361-5p, and miR-425-5p were significantly increased in HD patients as compared to that in controls. Further analysis showed that patients with higher Unified Huntington’s Disease Rating Scale total motor scores had significantly lower miR-122-5p levels. Increased miR-100-5p levels and decreased miR-330-3p and miR-641 levels were associated with the Total Functional Capacity of patients with HD. The authors suggested that the circulating miRNA profile might be modified by disease progression, and thus, it could be a promising biomarker for monitoring disease status. Subsequently, Chang et al. (2017) examined miRNA expression levels in the peripheral leukocytes of 36 HD patients, eight pre-symptomatic HD carriers, and 28 healthy controls. They identified 13 candidate miRNAs and subsequently determined that miR-9^∗^ was significantly lower in HD patients than in the healthy controls. They did not find a significant correlation between the miR-9^∗^ levels and the Unified Huntington’s Disease Rating Scale; however, the potential implications of miR-9^∗^ as a biomarker in the peripheral leukocytes of HD patients require further investigation.

Advances in microarray analysis technology have provided prospects for the screening of miRNAs as biomarkers. Gaughwin et al. (2011) conducted microarray analysis to study differentially expressed miRNAs in *mHtt-Exon-1*-overexpressing cell lines. By comparing the expression levels of 56 candidate miRNAs, they concluded that miR-34b and miR-1285 were upregulated in m*HTT-Exon-1*-transfected human teratocarcinoma cell line, which is a model of *mHtt*-induced transcription is supported by immune complex 2 -positive, nuclear immunostaining and the reduction in pluripotent and neuron-specific transcript levels, including known *mHtt* targets (BDNF). The expression levels of these two miRNAs were studied in the plasma of patients with HD. The results showed that miR-34b levels decreased significantly in the plasma of pre-symptomatic HD patients as compared to that in the control group. In contrast, no significant differences were observed between the miR-1285 levels of the two groups. Differential miR-34b expression was verified in the HD cell model. On the basis of these results, the authors concluded that miR-34b represented a potential biomarker for HD that was stably expressed in the plasma and was detected before clinical symptoms are evident. However, their observations were based on a small patient cohort. Thus, more patients should be evaluated to determine the biomarker potential of miR-34b.

The PREDICT-HD study was a prospective observational study conducted for more than a decade at 32 international sites (September 2002–July 2014). All PREDICT-HD participants underwent genetic testing prior to enrollment in the study. Reed et al. (2018) collected CSF samples from 60 PREDICT-HD study participants (15 symptomatic, 30 pre-symptomatic, and 15 controls) to evaluate miRNA levels. A total of 2,081 miRNAs were detected, and six of these miRNAs (miR-135b-3p, miR-140-5p, miR-520f-3p, miR-3928-5p, miR-4317, and miR-8082) showed significantly increased levels in pre-symptomatic HD patients. The increase in the miRNA levels was positively correlated with the risk of HD onset, with the lowest miRNA levels in the control group and the highest levels in symptomatic HD patients. These levels tended to remain stable. Although the study of CSF biomarkers is not as extensive as that for peripheral biomarkers, Reed et al. (2018) suggested a clinical basis for using CSF miRNAs as biomarkers for HD patients, especially for the early diagnosis of pre-symptomatic HD patients.

Hoss et al. (2014, 2015a) systematically studied the relationship between miRNAs and the pathogenesis of HD and their potential use as HD biomarkers. In one study, the expression levels of four miRNAs in the plasma of the subjects (26 symptomatic, four asymptomatic, and eight controls) were measured, and the potential value of these miRNAs as HD biomarkers was evaluated. The plasma levels of miR-10b-5p and miR-486-5p increased significantly in symptomatic HD patients but did not change in asymptomatic patients. These results suggested that increased miR-10b-5p and miR-486-5p plasma levels might be clinically relevant for the diagnosis of HD (Hoss et al., 2015b).

The miRNAs were considered to be the first candidates for the next generation of biomarkers because they have some advantages over other candidates such as proteins and metabolites. First, miRNAs are more likely to lead to early diagnosis because of their upstream position in the regulatory cascade. Second, it is easier to discover new miRNAs as biomarkers through genomic tools such as oligonucleotide microarrays and deep sequencing, which provide higher throughput than mass spectrometry, the main tool for protein and metabolite biomarker identification. Third, low abundance miRNA biomarkers can be amplified and then detected in a clinical setting by real-time quantitative PCR (qPCR). qPCR has been used in FDA-approved clinical trials, but there is no equivalent method for detecting low abundance proteins or metabolites (Li and Kowdley, 2012). However, the use of circulating miRNAs as a diagnostic biomarker for HD still has the following problems that need to be addressed and resolved: (1) determination of the specificity of the circulating miRNA in the diagnosis of HD. The expression level of the circulating miRNA may be affected by the combination of multiple diseases in the same patient, which may affect the specificity of the diagnosis; (2) there is a lack of uniform and accurate detection method for circulating miRNA and a lack of internal reference of miRNA to correct the expression level, because the use of tissue-related internal reference to correct the expression level of circulating miRNA is not scientifically rigorous; (3) currently, the research on circulating miRNA as a diagnostic biomarker of HD is still in the exploratory stage, and the number of cases and sample types detected are limited. The sensitivity and specificity of the circulating miRNA that may be selected as a diagnostic biomarker of HD need further experimental verification; and (4) The pathological mechanism by which circulating miRNA is involved in HD progression remains unclear, and the reference range of the expression level of circulating miRNA under different pathological conditions (symptomatic or pre-symptomatic HD) is yet to be identified.

## The miRNAs-Based Therapeutic Strategies

The miRNA-based therapeutic strategies can be direct (i.e., increasing or decreasing specific miRNA levels to regulate target gene expression) or indirect (i.e., regulating target gene expression by miRNA activation or enhanced endogenous repair mechanisms in the brain). Despite a better understanding of the expression and function of miRNAs in HD, only a few miRNA-based strategies have been studied. Evers et al. (2018) delivered engineered miRNA targeting human *Htt* to mouse and minipig HD models through adeno-associated virus vector 5 (AAV5-miHTT) to verify the therapeutic effect of this technique. They found that intrathecal and bilateral thalamic injection of AAV5-miHTT could significantly improve the motor coordination of HD animals (Evers et al., 2018). Moreover, the survival time of the R6/2 HD mice treated with AAV5-miHTT was significantly prolonged. Additional experiments showed that changes in the striatum and cerebral cortex of the R6/2 HD mice in the AAV5-miHTT treatment group were significantly reduced, and neuronal dysfunction was significantly alleviated. These changes were accompanied by an improvement in HD symptoms. Similar results were obtained with transgenic HD minipigs (Evers et al., 2018). The long-term efficacy of AAV5-miHTT was evaluated by injecting AAV5-miHTT into the striatum of Q175 HD mice. The effect of AAV5-miHTT therapy was dose-dependent and reduced the Htt protein by inhibiting the formation of mHTT aggregates in the striatum and cortex. After 8 weeks of AAV5-miHTT therapy, the dyskinesia of HD transgenic mice was significantly improved, and the median survival time of the treated mice was prolonged by 4 weeks. Indeed, studies confirmed that AAV5-miHTT could reduce aggregate formation, prevent neuronal dysfunction, and alleviate HD-like symptoms (Spronck et al., 2019). By injecting AAV5-miHTT into the bilateral striatum of an HD rat model, Miniarikova et al. (2017) showed that AAV5-miHTT could effectively inhibit mHTT mRNA and almost completely prevent the formation of mHTT. The authors believed that the structure of AAV5-miHTT is the most favorable factor in this approach; this is because AAV5 delivery of miRNA-mediated reduction of HTT did not activate microglia or astrocytes, indicating that the AAV5 vector or the therapeutic precursor sequence does not induce an immune response (Miniarikova et al., 2017). Recently, similar HD animal model experiments have also yielded consistent results, further confirming the promising prospects of intrastriatal miRNA-based gene therapy (Spronck et al., 2021; Vallès et al., 2021). However, when designing an HTT-lowering therapy that simultaneously reduces mHTT and wtHTT proteins in a non-selective manner, the role of wtHTT needs to be considered to achieve a balance between the benefits of reducing mHTT and maintaining sufficient wtHTT levels to perform its normal cellular functions. In addition, the administration of AAV5-miHTT is also a challenge when unexpected side effects occur. Therefore, although strong preclinical efficacy is required, safety is more important (Miniarikova et al., 2016; Caron et al., 2020; Evers and Konstantinova, 2020). Such a safety package should include toxicology studies conducted under good laboratory practice as well as immunogenicity and human sequence-specific miRNA off-target analysis. The key difference in this single treatment is that a more invasive injection method is to inject directly into the areas known to be most affected by the disease, the striatum. Although attempts to target these downstream pathways for therapeutic benefit have not been successful (Travessa et al., 2017), further investigation on miRNA dysregulation and the pathogenesis of HD and the development of safe and effective RNA interference drugs remain a potential therapeutic approach for HD.

## Conclusion

Emerging studies have confirmed a role of dysregulated miRNAs in the pathogenesis of HD, some of which have been consistently identified as HD-specific. As biomarkers, miRNAs have great potential for diagnosing HD and for monitoring disease progression and treatment response. Further studies are required to better understand their roles in physiological and pathological conditions and to verify their effectiveness as non-invasive biomarkers. With the advent of clinical trials, therapeutic miRNA gene intervention methods are likely to change the course of this neurodegenerative disease.

## Author Contributions

XD searched the literature and wrote the manuscript. SC critically revised the manuscript. Both authors have made a substantial, direct and intellectual contribution to the work, and have approved it for publication.

## Conflict of Interest

The authors declare that the research was conducted in the absence of any commercial or financial relationships that could be construed as a potential conflict of interest.

## Publisher’s Note

All claims expressed in this article are solely those of the authors and do not necessarily represent those of their affiliated organizations, or those of the publisher, the editors and the reviewers. Any product that may be evaluated in this article, or claim that may be made by its manufacturer, is not guaranteed or endorsed by the publisher.
